# Manipulating polymer composition to create low-cost, high-fidelity sensors for indoor CO_2_ monitoring

**DOI:** 10.1038/s41598-021-92181-4

**Published:** 2021-06-24

**Authors:** Zachary A. Siefker, John N. Hodul, Xikang Zhao, Nikhil Bajaj, Kelly M. Brayton, Carsten Flores-Hansen, Wenchao Zhao, George T.-C. Chiu, James E. Braun, Jeffrey F. Rhoads, Bryan W. Boudouris

**Affiliations:** 1 School of Mechanical Engineering, Purdue University, West Lafayette, IN 47907 USA; 2 Ray W. Herrick Laboratories, Purdue University, West Lafayette, IN 47907 USA; 3 Department of Chemistry, Purdue University, West Lafayette, IN 47907 USA; 4 Charles D. Davidson School of Chemical Engineering, Purdue University, West Lafayette, IN 47907 USA; 5 Birck Nanotechnology Center, Purdue University, West Lafayette, IN 47907 USA

**Keywords:** Sensors and biosensors, Polymers

## Abstract

Carbon dioxide (CO_2_) has been linked to many deleterious health effects, and it has also been used as a proxy for building occupancy measurements. These applications have created a need for low-cost and low-power CO_2_ sensors that can be seamlessly incorporated into existing buildings. We report a resonant mass sensor coated with a solution-processable polymer blend of poly(ethylene oxide) (PEO) and poly(ethyleneimine) (PEI) for the detection of CO_2_ across multiple use conditions. Controlling the polymer blend composition and nanostructure enabled better transport of the analyte gas into the sensing layer, which allowed for significantly enhanced CO_2_ sensing relative to the state of the art. Moreover, the hydrophilic nature of PEO resulted in water uptake, which provided for higher sensing sensitivity at elevated humidity conditions. Therefore, this key integration of materials and resonant sensor platform could be a potential solution in the future for CO_2_ monitoring in smart infrastructure.

## Introduction

It is estimated that the average American and European spends nearly 90%^1–3^ of their time indoors, and consequently, CO_2_ concentrations in buildings can range from those seen in outdoor environmental conditions (i.e., ~ 400 ppm) to 3000 ppm, depending on the occupancy of the confined space^4,5^. Moreover, CO_2_ is regarded as a toxic contaminant with an OSHA time-weighted average (TWA) exposure limit of 5000 ppm over an 8-h workday or a short-term exposure limit of 15,000–30,000 ppm over 15 min^6^. Importantly, CO_2_ exposure at as low of a concentration as 1000 ppm negatively affects cognitive performance, including decision making and problem resolution^7–9^. Thus, appropriate indoor air quality monitoring and building ventilation systems, equipped with low-cost, high-fidelity CO_2_ sensors, are required. However, many current sensing technologies suffer from limitations and drawbacks, such as arduous device fabrication techniques or high power consumption, which limits their practical and widespread implementation in residential, commercial, and industrial settings. Thus, there is a critical need for a low-cost, low-power CO_2_ sensor that is easy to manufacture and can be incorporated into existing buildings to upgrade these structures into “smart buildings” in a seamless manner.

State-of-the-art CO_2_ detection methods have relied primarily on gas chromatography and spectroscopy methods, which are typically high-cost and require large instrumentation footprints^10–13^. In particular, there has been a large push to use infrared (IR) spectroscopy to detect CO_2_ gas. Typically, CO_2_ infrared sensors are nondispersive infrared (NDIR) sensors where a broadband lamp source and an optical filter are used to select a narrow band in the spectral region that overlaps with the absorption region of the gas of interest. However, NDIR detection of CO_2_ is limited by spectral interference, a high detection limit, and interference from water vapor^14^. In fact, even many new NDIR CO_2_ sensors have readings that deviate from actual CO_2_ concentrations by more than 75 ppm^15,16^ and potentially even greater error if not regularly calibrated^17^. Additionally, the infrared light inherent to NDIR sensors, often results in bulky instrument size due to the length of the light path (> 1 cm) and high power consumption (> 200 mW), which limits their use in embedded applications, such as internet-of-thing-based (IoT-based) smart buildings. Recently there has been work in creating a handheld, low-power (i.e., < 1 W), and sensitive (i.e., 50 ppm) CO_2_ NDIR sensor capable of performing breath analysis^18^. Though intriguing, this device is not being manufactured for indoor air quality monitoring, and therefore lags in areas of testing and performance typical of indoor environments^18^. Thus, current NDIR CO_2_ sensors do not meet the power, size, and selectivity metrics necessary for a practically scalable sensor that can meet the demands of smart building technologies suitable for the indoor monitoring of CO_2_.

Conversely, microelectromechanical systems-based (MEMS-based) resonant mass sensors are a promising sensor paradigm for this application space as they exhibit high performance metrics in gas detection due to their compact size, low cost, low power, fast response times, and high sensitivity^19–23^. Importantly, when microresonators are functionalized with specific surface chemistries, target analytes non-covalently bind, or otherwise chemo-mechanically interact, with the sensor. This change in mass on the surface induces a shift in the resonant frequency of the device, which can be readily and precisely quantified. In fact, cantilevered-type resonant mass sensors are sensitive enough to detect bacteria and a single virus in the air^24,25^. Therefore, MEMS-based devices are an ideal platform for smart building integration if they can be functionalized with the appropriate and selective chemistry in a low-cost, high-throughput manner^26^. Given this potential, we fabricated a resonant mass sensor with a solution-processable polymer blend of poly(ethylene oxide) (PEO) and poly(ethyleneimine) (PEI) coated atop the resonant platform. The selection of these two materials was rather straightforward. First, PEI contains multiple amine groups that have been shown to effectively perform reversible acid–base reactions with CO_2_^27–30^. However, due to the viscous nature of PEI, diffusion of the CO_2_ into the material to perform such reactions is limited. Thus, we employed a hydrophilic, semicrystalline polymer, PEO^31^, in the blend with PEI to enhance the sensitivity and response rate of CO_2_ uptake. The PEO has two roles when being incorporated into the PEI. First, it disrupts intrachain and interchain PEI entanglement at the molecular level and the surface morphology at the nanoscale level. Both of these positive disruptions facilitate increased interactions between accessible amines and CO_2_. Second, the hydrophilic nature of PEO attracts water into the blended thin film; in turn, this water converts the reversible acid–base formed carbamates (i.e., the product of CO_2_ reacting with primary and secondary amines) into bicarbonates freeing amines to enhance the adsorption and uptake of CO_2_. This critical addition of the PEO moiety allows for the macromolecular blend to have selective and significantly enhanced detection of CO_2_ relative to previous efforts. This synergetic chemical blending and subsequent polymer processing, in combination with the resonant mass sensor platform, allows for the fabrication of a low-cost and effective CO_2_ sensor. In comparison to other CO_2_ sensing counterparts, this sensor is compact, with a footprint of < 25 mm^2^, and offers high sensitivity (i.e., at a detection limit of CO_2_ as low as 5 ppm). Moreover, these sensors detect CO_2_ selectively over other polar compounds (e.g., methanol and acetone), as well as non-polar compounds (e.g. xylene and propane). Thus, this sensing platform offers an easily functionalized, low-cost, low-power, multi-channel sensing array capable of quick and reliable detection.

## Results

### Sensor response and dynamic range

**Figure 1:**
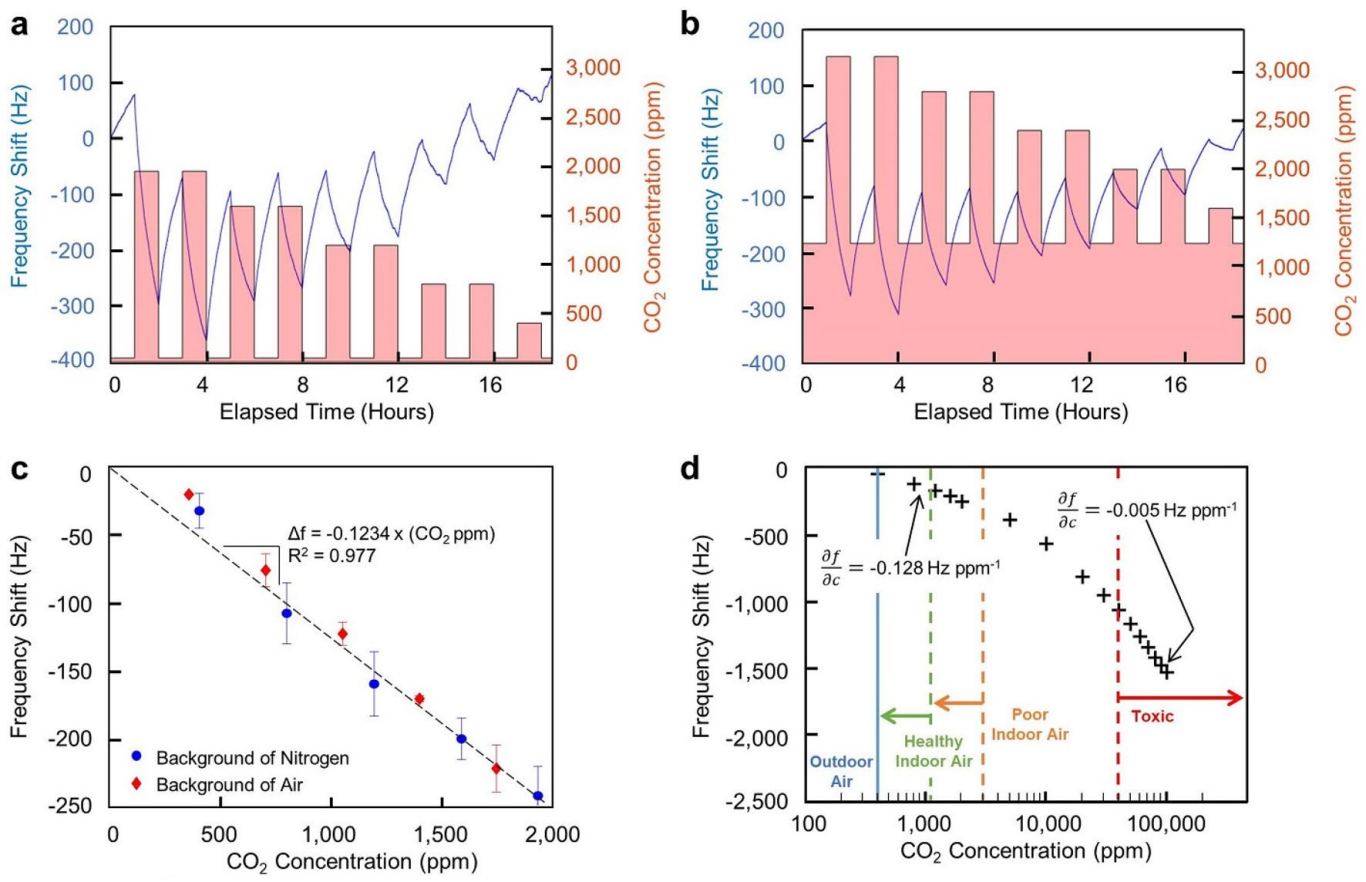
Sensor response to CO_2_ shown as a frequency shift of the resonant element. (**a**) Sensor response to CO_2_ over time with a background of nitrogen. The resonant frequency shift response is indicated by the blue line (left vertical axis), and the red bars indicate when CO_2_ is present (right vertical axis). (**b**) Sensor response to CO_2_ over time with a background of air. The resonant frequency shift response is indicated by the blue line (left vertical axis), and the red bars indicate when CO_2_ is present (right vertical axis). The use of air brought the baseline CO_2_ concentration to 1200 ppm. (**c**) Total frequency shift of the device after 1 h of CO_2_ at specified concentrations. The average of 8 responses is shown with error bars representing one standard deviation. Regardless of the baseline conditions, a similar linear response is obtained by the sensor, as demonstrated by the linear regression fit. (**d**) The dynamic range of the sensor is shown by plotting the resonant frequency shift in response to 1 h of CO_2_ in a background of nitrogen. For comparison, outdoor air CO_2_ concentrations^35^ are indicated by the solid blue line, healthy indoor air CO_2_ levels^32^ are indicated by the dashed green line, poor indoor air CO_2_ levels^32,36^ are indicated by the dashed orange line, and toxic CO_2_ levels^37^ are indicated by the dashed red line. Additionally, the sensitivity (indicated by the change in frequency per change in CO_2_ concentration, $\frac{\partial f}{\partial c}$) is shown at both low and high concentrations.

Resonant mass sensors functionalized with a PEI-PEO polymer blend were able to detect CO_2_ across an extended range of concentrations relevant to indoor air quality monitoring (Fig. 1). Critically, these sensors demonstrated a highly linear response at CO_2_ concentrations relevant to the targeted application of buildings. This range is roughly defined by a lower bound of 400 ppm (i.e., the concentration that is typical for air outdoors, located in unoccupied spaces) and an upper bound of 2000 ppm. A building with well-controlled ventilation will have CO_2_ concentrations around 1100 ppm, which is the central region of interest^32^. Fig. 1a–c highlight sensor responses at CO_2_ concentrations that are relevant to indoor environments. These data demonstrate sensor performance in a background of nitrogen and a background of air (i.e., ~ 78% nitrogen, ~ 21% oxygen, and 0.12% carbon dioxide). In both the nitrogen and air backgrounds, the sensor response was linear and proportional to the increase in CO_2_ concentrations above the background concentrations with an interpolated sensitivity of approximately 0.12 Hz ppm^−1^ CO_2_ (Fig. 1c). However, these responses were determined for 1 h pulses of CO_2_ which was often not long enough for the functional material to fully saturate with carbon dioxide. This delayed response and recovery time may prove problematic in some applications. In such cases, a thinner film of the functional material may be used such that it saturates with CO_2_ in less time. However, this will be done at the expense of increasing the limit of detection which may be interpolated based on a 1 Hz frequency resolution. Thus, optimization of this point will be specific to the end-use application. Further, the desorption of CO_2_ occurs at a slower rate than adsorption, resulting in the drifting baseline in the time series data (Fig. 1a,b). The time series data (Fig. 1a,b) show the resonators decreasing in frequency as CO_2_ is adsorbed on top of the resonator. The added CO_2_ mass on the resonator decreases its resonant frequency, as described, for example, by the Sauerbrey equation^33,34^. The magnitude of the frequency shift is greater at higher concentrations due to an increased amount of CO_2_ adsorbing onto the resonator. Thus, the change in CO_2_ concentration induces a clear shift in resonant frequency of the sensor.

Moreover, this sensor has a large dynamic range and can detect CO_2_ at concentrations well beyond what is relevant to indoor air monitoring (Fig. 1d). Unlike the relatively linear response demonstrated below 2000 ppm CO_2_, when the sensor is exposed to larger concentrations, a nonlinear response is apparent as the frequency shift per ppm of CO_2_ decreases asymptotically. This is consistent with a Langmuir sorption model where the rate of adsorption decreases as the available surface binding sites are filled^38^. Nevertheless, this sensor can discriminate CO_2_ concentrations well beyond toxic levels for human occupancy^37^.

### Performance in indoor environments

**Figure 2:**
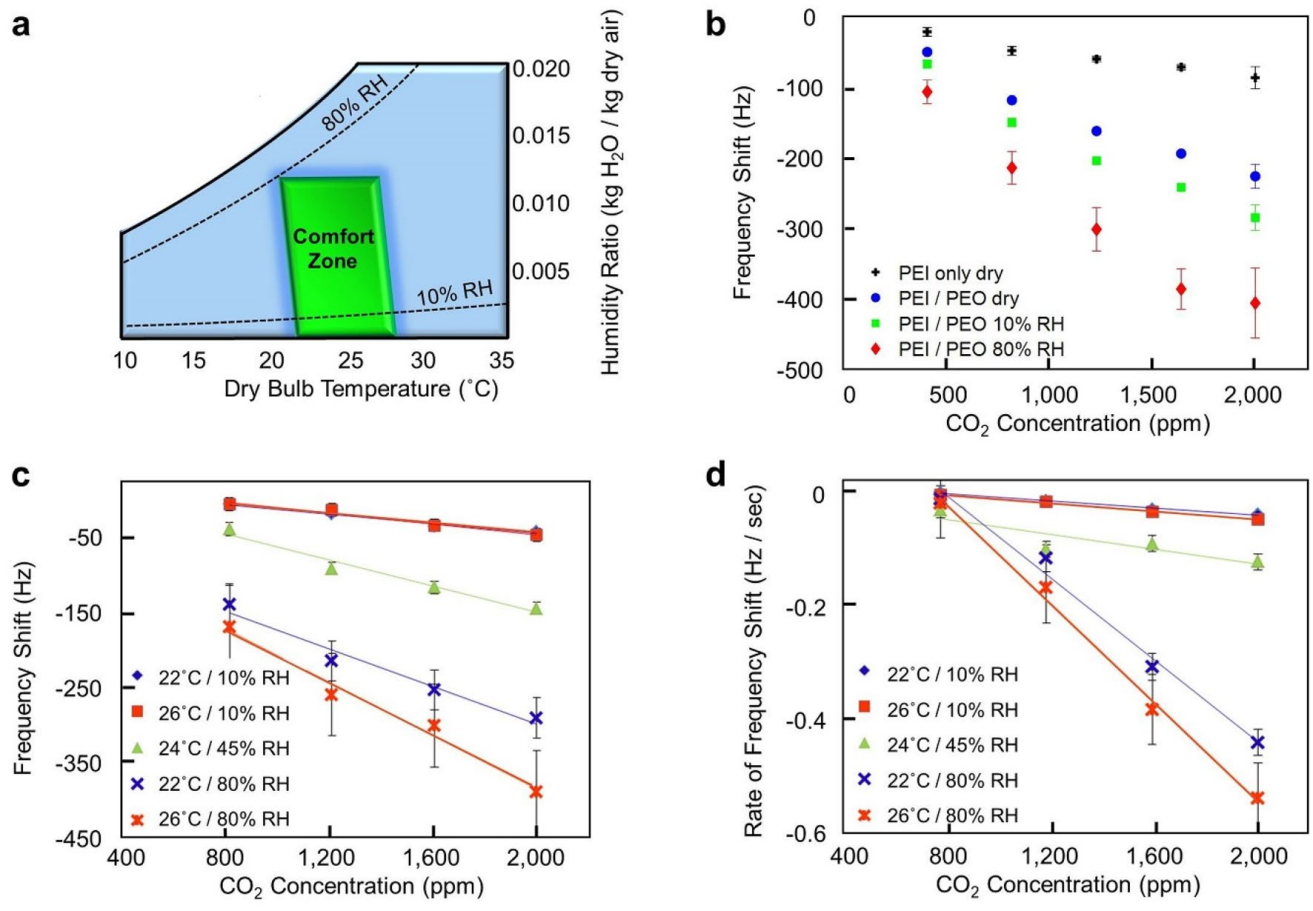
(**a**) Psychrometric chart defining the ‘Comfort Zone’ air temperature and humidity range, as defined by the engineering association, ASHRAE. Testing was performed across this region to simulate environmental conditions expected in high performance buildings. (**b**) Frequency response of devices functionalized with PEI only, and a 3:1 PEO:PEI blend ranging from 0 to 80% relative humidity (RH). Tests were performed holding temperature at 24 °C. The average of 8 responses is shown with error bars representing one standard deviation. (**c**) Sensor response to CO_2_ while varying temperature from 22 to 26 °C and relative humidity from 10 to 80% to cover the indoor air comfort zones^39^. The average of 8 responses is shown with error bars representing one standard deviation. A baseline condition of 400 ppm CO_2_ in nitrogen was used to simulate outdoor air conditions and a linear response is obtained at each condition, as demonstrated by the linear regression fits. (**d**) The rate of change in frequency shift after each increase in CO_2_ concentration from a background of 400 ppm. The average of 8 responses is shown with error bars representing one standard deviation. A similar linear response is obtained at each condition, as demonstrated by the linear regression fits.

Even in a relatively well-controlled, indoor environment, air conditions can drastically vary in both temperature and humidity level. Thus, it is important to account for these variables and demonstrate sensor performance across a span of potential environmental conditions. A ‘comfort zone’, which describes indoor air conditions that most people find comfortable, has been defined by the engineering society ASHRAE^39^. This region is outlined on the psychrometric chart (Fig. 2a) based on indoor air temperatures and humidity levels. Using the comfort zone as a guide, a test region was developed spanning approximately 22–26 °C and 0–80% relative humidity (at a given temperature), and testing was performed near the extremes of this region, as well as near the center.

Previously, PEI has been used to detect and capture CO_2_^27,28,40–42^. This is because CO_2_ reacts with the primary and secondary amines of PEI readily to form carbamates that can be stabilized by other primary amines or with water and create bicarbonate ions^29^. When PEI is mixed with PEO and supported onto mesoporous oxides, PEI is a powerful adsorbent of CO_2_^43,44^. However, few studies have evaluated the adsorption of CO_2_ onto blended films of PEO and PEI without a mesoporous substrate for the purpose of measurement under indoor air conditions^45,46^. It must be noted that in these examples of PEO and PEI polymer blend films, that these films were fabricated with graphene-based materials. In our case, without any graphene additives, the PEO-PEI blend enhanced the frequency response and sensitivity of the sensor to CO_2_ (Fig. 2b, Table S1). Under dry air conditions (i.e., 0% RH) the frequency response of the polymer blend is more than twice that of PEI alone. Additionally, when increasing the background relative humidity level to 10% and then to 80% at a constant temperature, the frequency shift response to CO_2_ is nearly tripled and then quadrupled, respectively, relative to the resonator functionalized with PEI only. Furthermore, the PEO and PEI blends have shown promising performances in both elevated temperature and elevated humidity conditions, simultaneously (Fig. 2c,d). However, the addition of the hydrophilic PEO chemistry induces an inherent cross-sensitivity to water. Therefore, commercial implementation of this sensing device would require humidity compensation. However, this is a commonly-monitored metric in commercial HVAC systems. Thus, only simple, complementary circuitry would be required to account for the presence of humidity, and this could easily be manufactured within the same device as our sensors.

### Sensor selectivity

**Figure 3:**
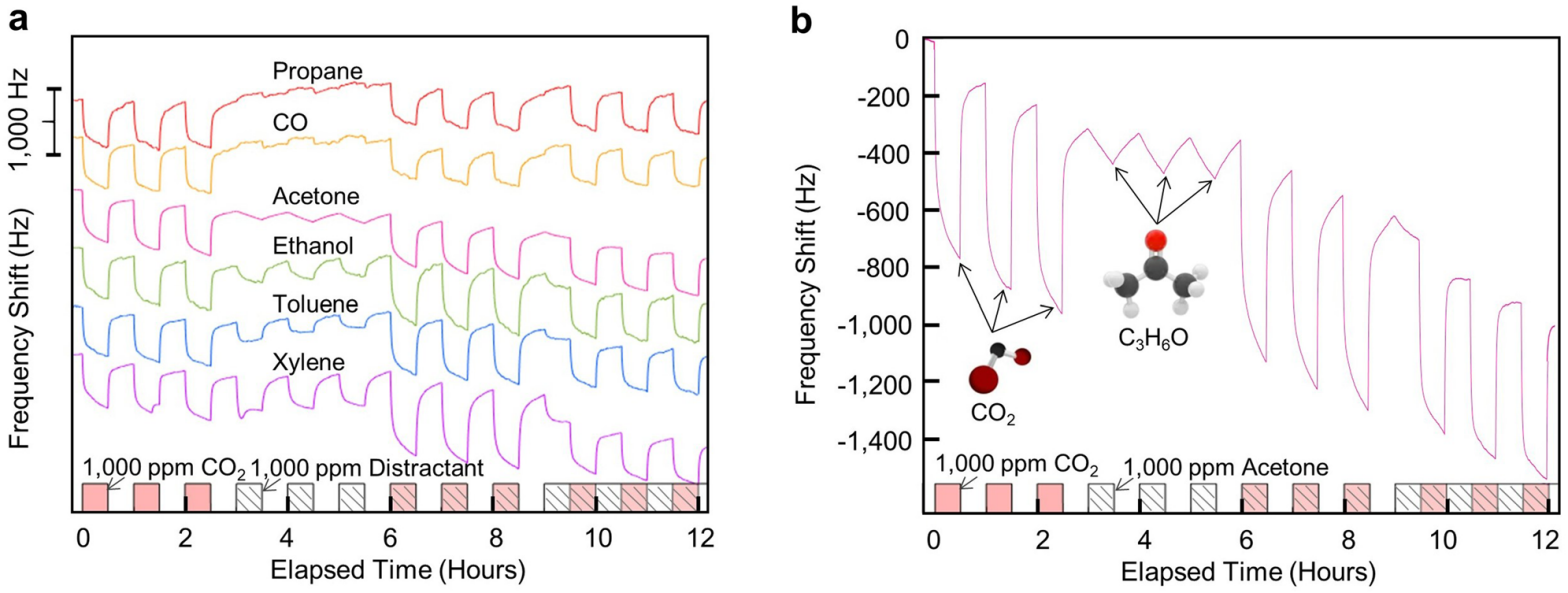
Sensor response to 1000 ppm pulses of CO_2_ and various distractants. (**a**) Frequency shift responses of a sensor in the presence of various interfering gas analytes. (**b**) Zoomed frequency shift response of a sensor in response to CO_2_ in the presence of acetone. The time series data shows 30 min pulses of CO_2_ with a background of nitrogen, followed by pulses of acetone, pulses of CO_2_ and acetone together, and finally pulses of CO_2_ with a constant background of acetone.

These sensors showed relative selectivity to CO_2_ when interfering gases (i.e., distractant gases) were introduced into the chamber (Fig. 3a). Previously, PEI-based materials have shown responses to other analytes^47^. Therefore, selectivity testing was performed with analytes that could be present in an interior location. Specifically, these tests were performed with a variety of confounding species including propane, carbon monoxide, acetone, ethanol, toluene, and xylene. Though not an exhaustive list, each of these species were carefully selected to cover a broad range of functional chemical groups (i.e., alkyl hydrocarbons, ketones, alcohols, and aromatic hydrocarbon) typically found in interior locations. Each test alternated between 30 min pulses of analyte gases and a nitrogen purge of the test chamber. CO_2_ and the selected interfering gases were each introduced at concentrations of 1000 ppm for comparison. It should be noted that 1000 ppm is a common CO_2_ level for indoor environments; however, for the distractants considered, this concentration is well above what is considered safe^36^. Thus, these distractant gases tested are on the extreme side of what could potentially confound the sensors under practical operating conditions. Tests were performed to show a CO_2_ pulsed alone, the distractant vapor pulsed alone, CO_2_ and the distractant pulsed together, and CO_2_ pulsed with a background distractant level. Testing in this manner allowed for a direct comparison of sensor response to CO_2_ and the distractant, as well as any interacting effects on the sensor when CO_2_ and the distractant are presented together. Cross-sensitivity is apparent; however, for all of the distractants tested, the sensor had a distinguishable response to CO_2_ and a lesser response (if any) to the distractant vapor. Acetone, for example, induced an average shift of 128 Hz during a 30 min pulse, whereas, CO_2_ induced an average shift of 734 Hz for the same time period (Fig. 3b). Thus, the influence of acetone, at a relatively high concentration for indoor environments, was nearly 6 times less than the sensor’s response to CO_2_ at the same concentration.

### Polymer film properties impact CO_2_ detection

The optimal and reliable detection of CO_2_ using a 3:1 PEO:PEI blend film is due to the ability of the semi-crystalline nature of PEO to disrupt the intermolecular interactions of the hyperbranched PEI. PEI, a polymer with many amine branches, ranging from primary to tertiary amines, is an amorphous material that has a relatively low glass transition temperature (i.e., in our hands, it was – 50 °C for a PEI molecular weight of 25 kg mol^−1^). Thus, branched PEI at room temperature exists as a viscous liquid due to the strong non-covalent interchain amine interactions and polymer entanglements^48^. PEI, when drop cast onto a substrate and dried under vacuum, remains a uniform polymer film with minor surface defects or disruptions (Figure S1a,b) on the PEI surface until another material or polymer is introduced into the PEI casting solution. Therefore, a pristine PEI thin film has difficulty detecting CO_2_ due to the high viscosity and nanostructural uniformity caused by the hyperbranched amine interchain interactions of a PEI film (Fig. 2b), which can hydrogen bond and become highly entangled with each other. These entangled amines, many of which are primary amines, prevent the ready diffusion of CO_2_ into the material to interact, which ultimately limits the CO_2_ uptake and subsequent sensor response.

**Figure 4:**
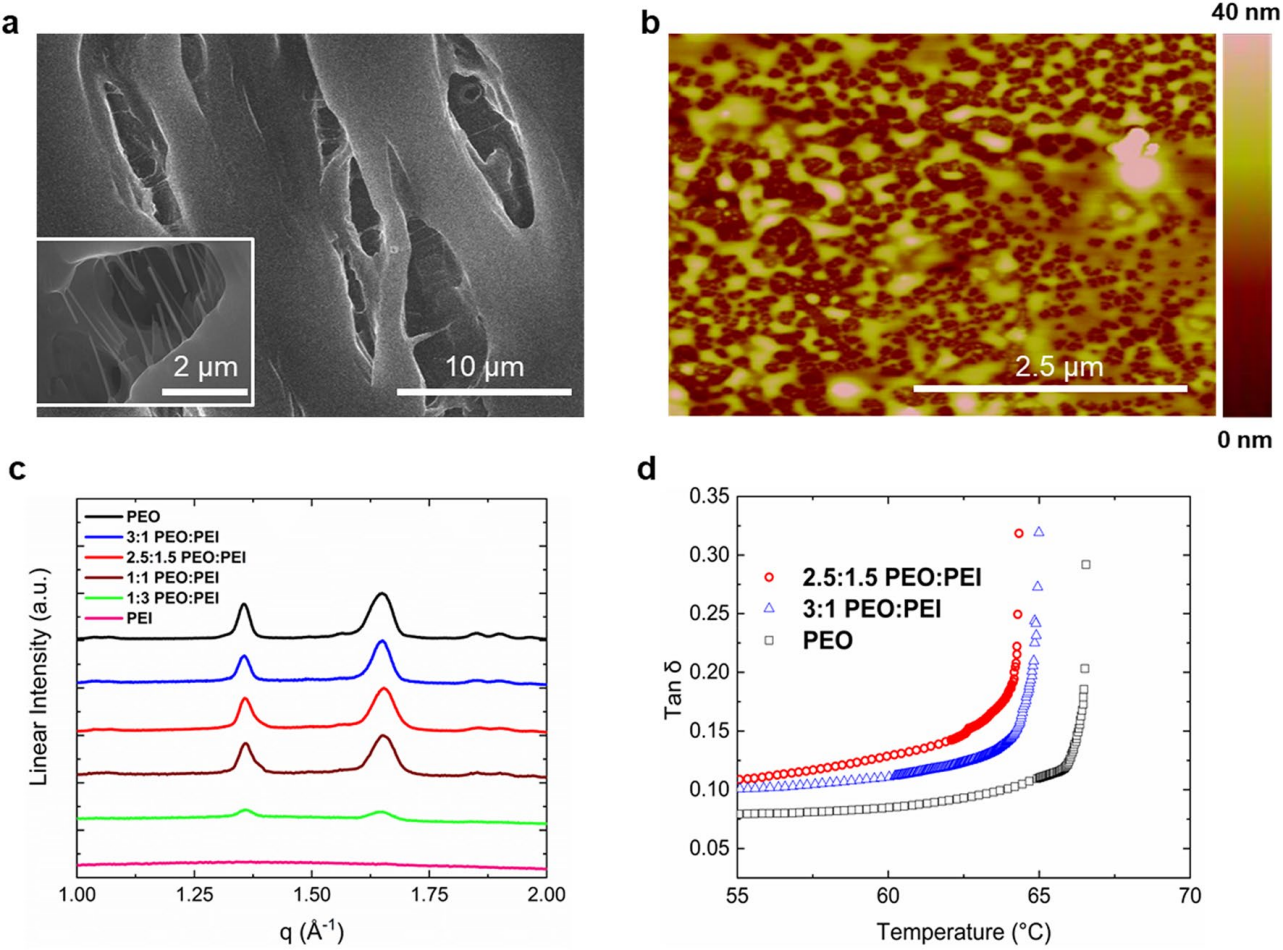
(**a**) SEM images of a 3:1 PEO:PEI blend film. Both images shown are of the same film but in different sections and locations. (**b**) AFM images of a 3:1 PEO:PEI blend films. (**c**) XRD patterns of PEO, 3:1 PEO:PEI blend, 2.5:1.5 PEO:PEI blend, 1:1 PEO:PEI blend, 1:3 PEO:PEI blend, and PEI only films. (**d**) DMA of a 2.5:1.5 PEO:PEI blend, 3:1 PEO:PEI blend, and PEO-only films.

When PEO is blended in a 3:1 ratio with PEI, surface features and nanoscale disruptions in the blended polymer film were observed (Fig. 4a,b). This surface segregation also occurred to a lesser degree in the 1:1 PEO:PEI blend film (Figure S1c,d). These defects and disruptions in the PEI film are indicative of altering macromolecular interactions when PEO is incorporated into a PEI matrix. This macromolecular rearrangement promotes CO_2_ adsorption because CO_2_ can now diffuse into the PEI material and interact readily with accessible primary and secondary amines which were not accessible prior to the addition of PEO. Moreover, when incorporating the PEO into the PEI matrix there was an observed crystal structure as seen in X-ray diffraction (XRD) (Fig. 4c) with characteristics reflections at q = 1.3 Å^−1^ and q = 1.65 Å^−1^, which is consistent with the monoclinic crystal structure of PEO^49,50^.

When PEO is added to the amorphous PEI, PEO tries to maintain order, and this ordering of PEO disrupts the PEI entanglements. In turn, this reduces the side chain amines from interacting with other interchain amines. As a result, there is polymer phase separation and surface morphology alterations in the film. When these alterations occur, CO_2_ was transported into the PEI matrix in a more robust manner such that larger uptake occurred. Thus, as seen in a 0% relative humidity environment, the PEO:PEI blended film can interact better with CO_2_ than PEI alone (Fig. 2b). This surface morphology alteration agrees with previous work with similar blends when the PEO molecules physically shielded the PEI chains from one another to reduce individual PEI-PEI chain interactions^43^. This PEO-induced physical disruption of interchain PEI amines from one another is in direct agreement that intrachain CO_2_ adsorption events (i.e., rather than interchain) are a result of the PEO causing less PEI branched amine interchain interactions resulting in less diffusional limitations and more reactivity between CO_2_ and primary and secondary amines.

Furthermore, dynamic mechanical analysis (DMA) showed that a pure PEO film has a decreased tan δ maximum value of 0.28 in comparison to films containing amorphous PEI. This increased storage modulus in PEO reinforces our observations in the XRD data showing that PEO maintains an ordered structure (Fig. 4d). Unlike PEI, which behaves as a highly viscous liquid with many entangled amines, PEO has a low tan δ due to a larger storage modulus due to the crystalline order in the material. When PEO is added into the PEI material, at 2.5:1.5 and 3:1 PEO:PEI blend films the tan δ maximum was 0.32. As the ratio of PEO to PEI increases, the tan δ signal decreased due to the physical disruptions of PEO solvating and shielding branched PEI amines as PEO tries to maintain its crystal structure. As a result, this orders the polymer matrix, creates less amine entanglements, and increased the storage modulus in the blended material. This ordering of the PEI by PEO at the 3:1 PEO:PEI ratio produces a tan δ maximum in the material capable of interacting with CO_2_. More specifically, this tan δ maximum caused by the PEO allows for less amine entanglements which causes more surface area for CO_2_ to adsorb into the material to interact with PEI. Thus, more CO_2_ can interact with PEI intrachain amines and yield a better response on the sensor device.

### The hydrophilic properties of PEO further enhance CO_2_ detection

**Figure 5:**
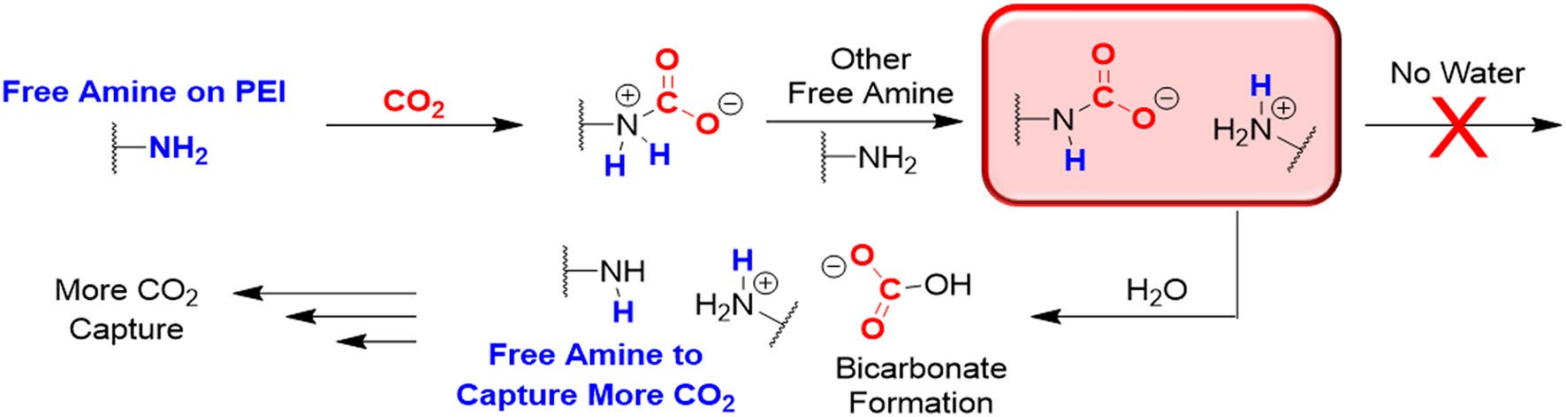
Proposed mechanisms of CO_2_ capture by PEI with and without the prescence of water. The intermediate step indicated in red is the key step in this reaction process. Without the presence of water the reaction does not proceed and the amine does not become accessible. With the presence of water the reaction can proceed forward due to a free amine which can capture more CO_2_.

In addition to providing structural changes in the PEI matrix, PEO facilitates water uptake into the polymer matrix, and this allows for more reversible acid–base reactions to occur between the PEI amines and CO_2_. When the 3:1 and 1:1 PEO:PEI blend films were exposed to a humid environment and had their Fourier Transform Infrared Spectroscopy (FTIR) spectra acquired, there was observed water uptake into the blend films (Figure S2). This water uptake in the 3:1 PEO:PEI films is important when detecting CO_2_ because these films showed a frequency shift response to CO_2_ that was significantly enhanced relative to the resonator functionalized with PEI only (Fig. 2b–d). PEI is a highly branched polymer with primary, secondary and tertiary amino groups, and these different chemical environments interact with CO_2_ in distinct manners^51,52^. That is, primary and secondary amino groups can directly react with CO_2_ to form carbamate groups by a reversible acid–base reaction. More specifically, direct nucleophilic attack on a free CO_2_ by a primary or secondary amine forms a zwitterion, which rapidly rearranges to carbamic acid via intramolecular proton transfer. In the presence of another free amine, which now acts as a Brønsted base, the carbamic acid may be converted into a carbamate via intermolecular proton transfer. Thus, these carbamates are stabilized by another amine that becomes protonated to form an ammonium ion. Under dry conditions the reaction stops here; however, the availability of water can further convert the carbamate into stable bicarbonate (Fig. 5). As a result, this frees an amine that then can react with more available CO_2_ gas. Tertiary amines do not directly react with CO_2_ without water. However, there is the possibility that water and CO_2_ can form carbonic acid which can be deprotonated by all of the types of substituted amines to form bicarbonate as well. In this alternative route, carbonic acid groups are typically deprotonated at neutral pH by a single amine (i.e., any substitution) instead of multiple amines. Due to a single amine being involved in the deprotonation on the carbonic acid this allows for another amine to be available to attack other carbonic acids that may be present. Regardless of which route is occurring (i.e., direct CO_2_ attack or deprotonation of carbonic acid), water does have a major impact on how the polymer matrix can interact with CO_2_. Therefore, PEO in addition to providing order to the PEI and increasing the accessibility of the amines to reversibly react with CO_2_ also increases the abundance of water in the polymer matrix when under humid conditions, and this increases the adsorption capacity of CO_2_ in the blended film.

## Discussion

We have presented a resonant mass sensor treated with a polymer blend of PEO and PEI for the sensitive and selective detection of CO_2_. The synergetic blending of PEO and PEI provided an enhanced materials platform for reversible sorption of CO_2_. This, combined with a MEMS-based resonant mass sensing platform, created an exceptional sensor for indoor CO_2_ monitoring. The small size and low power requirement of these sensors provides the necessary qualifications for wireless and distributed sensing in buildings^53–55^. Further, the readily accessible materials used to manufacture this sensor renders a device that is easy to procure at a low cost. Thus, this unique combination of well-known materials allows for a novel CO_2_ sensor that can be seamlessly integrated into smart building environments.

## Experimental methods

### Materials

All of the chemicals were purchased from Sigma-Aldrich, and they were used as received unless otherwise noted. The PEI utilized had a reported a weight average molecular weight of 25 kg mol^−1^. The PEO utilized was purchased from Alfa Aesar, and it had a reported weight average molecular weight of 100 kg mol^−1^. The methanol utilized to prepare the polymer samples was anhydrous grade and stored under nitrogen. Polished silicon dioxide substrates utilized for imaging were purchased from Silicon Valley Microelectronics.

### General methods

A Veeco Dimension 3100 Atomic Force Microscope (AFM) in tapping mode was utilized for AFM imaging. For these images, the polymer samples were fabricated by depositing 1.0 μL of a 1 mg mL^−1^ polymer blend in methanol on a polished silicon dioxide substrate. Then, the sample wafers were dried under vacuum (P ≤ 0.4 Torr) overnight to remove solvent. The images for the 3:1 PEO:PEI, 1:1 PEO:PEI, PEO-only, and PEI-only films were acquired using this protocol. A Hitachi S-4800 Field Emission scanning electron microscope (SEM) was utilized to image the PEO:PEI. For these images, 1.0 μL of a 1 mg mL^−1^ polymer blend in methanol solution were drop cast on polished silicon dioxide substrates and dried overnight under vacuum. All of the polymer films were then coated with 20 nm of conducting carbon using a SPI carbon sputter coater prior to imaging. The images for the 3:1 PEO:PEI, 1:1 PEO:PEI, PEO-only, and PEI-only films were acquired using this protocol. X-Ray diffraction (XRD) measurements were measured with a Rigaku Cu-Kα source (λ = 1.54056 Å) in parallel beam mode. These samples were acquired while under ambient conditions. The polymer film samples were fabricated using a 240 mg mL^−1^ solution of the polymer blend (by weight ratio) in methanol pipetted into a metal mold and then annealed at 80 °C while being pressed for at least 10 min. After pressing, these films were dried overnight under vacuum (P ≤ 0.4 Torr) prior to XRD analysis being performed. A Thermo-Nicolet Nexus Fourier Transform Infrared Spectroscopy (FTIR) with a KBr beam splitter with a 800 cm^−1^–4500 cm^−1^ spectra range was utilized for FTIR analysis of the PEO:PEI polymer films. The polymer films were fabricated utilizing the same protocol as discussed for XRD analysis. For DMA testing of the polymer films, a TA Instruments DMA Q800 with a film tension clamp was utilized. These experiments were conducted at a temperature ramp of 0.5 °C min^−1^ and at a constant frequency of 1 Hz. The polymer films were fabricated utilizing the same general protocol as discussed for XRD analysis.

### Device testing

**Figure 6:**
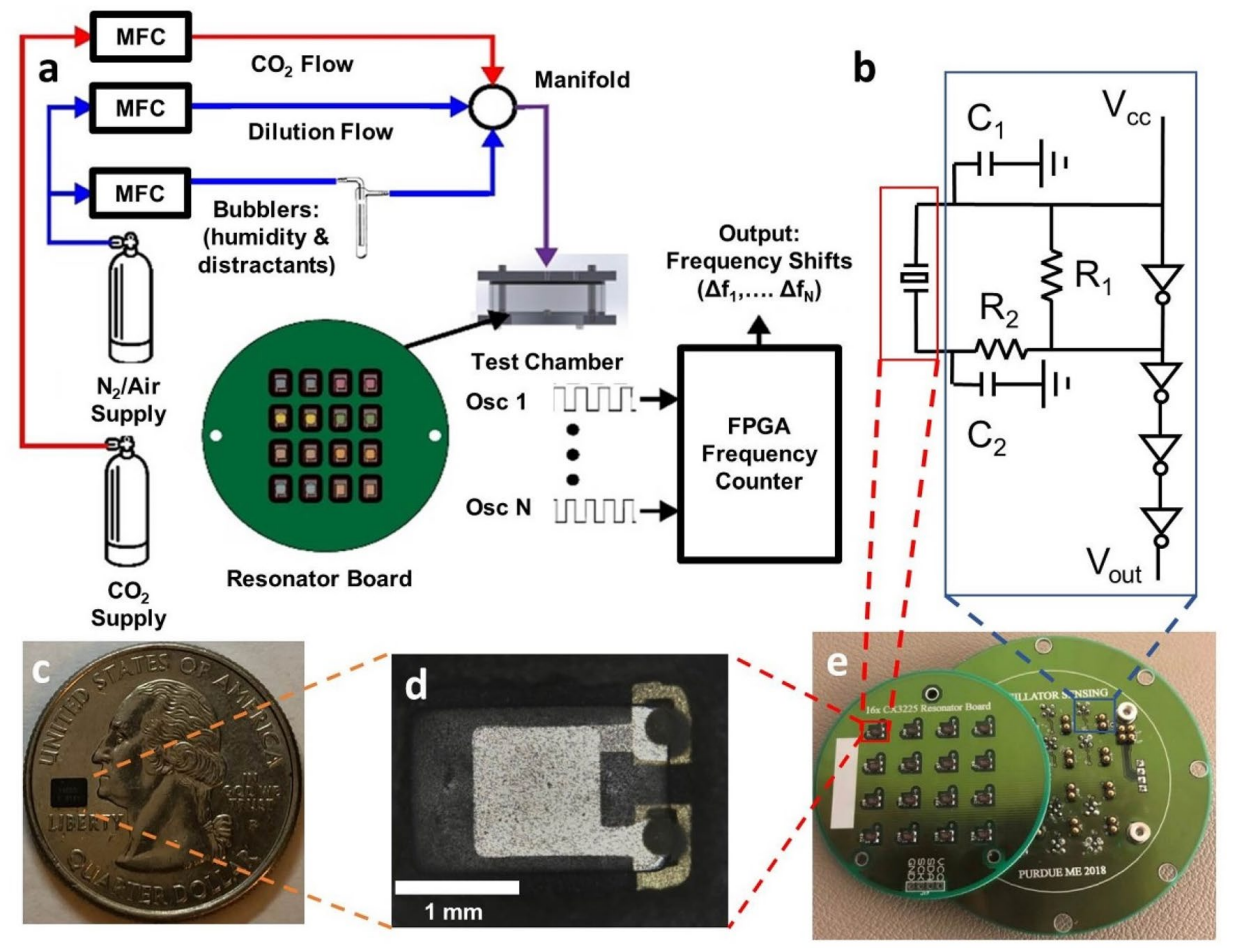
(**a**) Schematic of the gas distribution system used for sensor testing. Mass flow controllers (MFCs) modulated supply gases to the test chamber containing functionalized oscillators. Bubblers connected to a MFC were used to moderate distractant analytes and humidity levels inside the testing chamber. A frequency counter, executed on a MyRIO FPGA monitored the frequency of each oscillator in parallel. (**b**) A schematic of the Pierce oscillator used. The resonant element, outlined by the red box, is shown on the left of the circuit diagram. The remainder of the oscillator circuit, which is contained on the instrumentation board, is outlined by the blue box. This portion of the diagram contains two load capacitors (C_1_ and C_2_), a feedback resistor (R_1_), and an isolation resistor (R_2_). A series of inverters between the supply voltage (V_cc_) and the output voltage (V_out_) are used to square-off the oscillator output signal to facilitate frequency counting. (**c**) A single packaged resonant element shown on a US quarter for scale. (**d**) An exposed quartz crystal resonant element. (**e**) A resonant mass sensing system with 16 Pierce oscillators. A resonator board (left) containing 16 resonant elements is shown offset from the instrumentation board (right) which completes the Pierce oscillator circuit.

The testing protocols utilized in this work are similar to those previously reported^23,46,56^. Testing of the devices was performed using the experimental setup shown in Fig. 6a. Gas tanks of nitrogen, air, and carbon dioxide were connected to a series of mass flow controllers (MFC; MKS, 1179C) in parallel. A subset of nitrogen-supplied MFCs was connected to bubblers (ChemGlass, AF-0085) for introducing humidity or select vapor distractants (i.e., acetone, ethanol, toluene, and xylene). The gas lines converged to a mixing manifold, the output of which was connected directly to the test chamber inlet. A 95 mm diameter and 23 mm in height cylindrical aluminum testing chamber was used for evaluating the sensors. A 6.4 mm gas inlet port was centrally-located on the top of the test chamber and two 6.4 mm exhaust ports were located on opposite sides of the test chamber. The small chamber volume allowed for complete gas exchange in less than 1 min, facilitating quick sensor responses upon changing MFC flow rates.

Prior to sensor testing, the resonator board was attached to the instrumentation board. The test chamber was flushed with nitrogen or air to create an inert environment as the baseline for experimentation. Subsequently, the analyte gases were injected into the chamber to achieve the reported concentrations. A frequency counter was developed in LabVIEW to monitor the oscillation frequency of each oscillator with a 1 Hz resolution. To facilitate parallel monitoring, the frequency counter was synthesized and executed on an NI myRio Field Programmable Gate Array (FPGA). An FPGA consists of a collection of logic elements between which electrical paths can be created and allows for parallel computing. Thus, each of the 16 frequency counting loops could run simultaneously.

### Device instrumentation

The device instrumentation utilized in this work was previously reported^23,56^. An array of 16 Pierce oscillators was used as the sensing platform. Figure 6b shows the Pierce oscillator circuit, which consisted of an inverter, two load capacitors (C_1_ = 22 pF and C_2_ = 22 pF), one feedback resistor (R_1_ = 2 MΩ), one isolation resistor (R_2_ = 510 Ω), and a 16 MHz quartz crystal resonator (Kyocera Corp., CX3225). The crystal oscillator driver (Texas Instruments, SN74LVC1GX04) provided the circuit with the Pierce oscillator inverter as well as three additional inverters, which effectively squared the oscillator output signal. The hardware implementation of the oscillator circuit resulted in two printed circuit boards: (i) a board containing only an array of resonators and (ii) an instrumentation board containing the 16 sets of oscillator electronics with spring pin connectors in place of the resonators (Fig. 6e). As such, the resonator boards could be functionalized independent of the rest of the oscillator circuit and easily interchanged without incurring high component costs.

A single crystal resonator has a small footprint (8 mm^2^) and low power requirement (< 200 μW) during operation. Figure 6c shows a single packaged crystal resonator on top of a United States quarter, for scale. The small size and low power requirement of a single resonant sensor is particularly promising when considering wireless and distributed sensing in buildings^53–55^. The resonant element of the Kyocera CX3225 is shown in Fig. 6d, after the package cap has been removed. Functional materials were applied directly to this exposed element.

### Device functionalization

PEI and PEO were dissolved in methanol to generate a solution of 0.1% (by volume) of PEI and 0.3% (by volume) of PEO. Then, 1 μL of this solution was deposited onto each resonator. The resonator board was then placed under vacuum at 70 °C for 12 h to remove any residual methanol leaving behind a 3:1 PEO:PEI blend film (by weight). Unless otherwise specified, the devices utilized in the tests were all functionalized with the 3:1 PEO:PEI (by weight). Prior to testing, the resonator board was allowed to equilibrate back to room temperature.

## Supplementary Information

Supplementary Information.
