# Supplementary material for: Manipulating polymer composition to create low-cost, high-fidelity sensors for indoor CO2 monitoring
Source: Sci Rep. 2021 Jun 24;11:13237. doi: 10.1038/s41598-021-92181-4 (PMC8225849; doi:10.1038/s41598-021-92181-4)
Supplement: Supplementary file 1 — Supplementary Information. [file 41598_2021_92181_MOESM1_ESM.pdf]

Supplementary Materials

Manipulating Polymer Composition in Order to Create Low-Cost, High-Fidelity Sensors for Indoor CO_2 Monitoring

Zachary A. Siefker,[1,2] *John N. Hodul,*[3] *Xikang Zhao,*[4] *Nikhil Bajaj,*[1,2,5] *Kelly M. Brayton,*[4] *Carsten Flores-Hansen,*[3] *Wenchao Zhao,*[4] *George T.-C. Chiu,*[1,2,5] *James E. Braun,*[1,2] *Jeffrey F. Rhoads,*[1,2,5] *and Bryan W. Boudouris*[3,4]

[1] School of Mechanical Engineering, [2] Ray W. Herrick Laboratories, [3] Department of Chemistry, [4] Charles D. Davidson School of Chemical Engineering, and [5] Birck Nanotechnology Center, Purdue University, West Lafayette, Indiana 47907 United States

Table S1. A comparison of device performance using PEI and PEO.

	PEI dry	*3:1 PEO:PEI dry*	*3:1 PEO:PEI 10% RH*	*3:1 PEO:PEI 80% RH*
Interpolated sensitivity	-0.0502 Hz ppm^{-1} CO_2	-0.1271 Hz ppm^{-1} CO_2	-0.1590 Hz ppm^{-1} CO_2	-0.2349 Hz ppm^{-1} CO_2

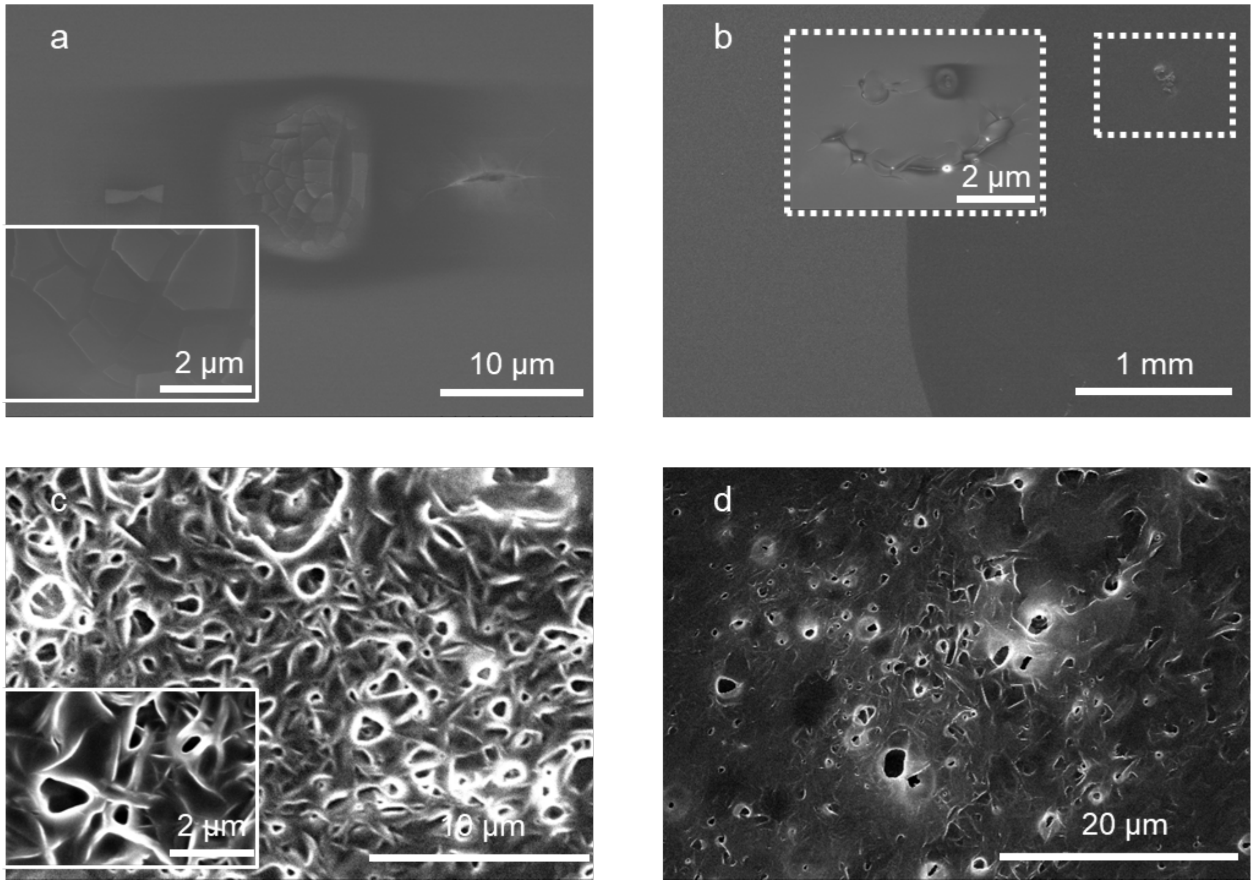

Figure S1. (a) and **(b)** SEM images of a PEI film after being drop cast onto a silicon wafer and dried for 12 h under vacuum. The two panels show the same film at two different magnification levels. **(c)** and **(d)** SEM images of a 1:1 PEO:PEI (by weight) blend film after being drop cast onto a silicon wafer and dried for 12 h under vacuum. The two panels show the same film at two different magnification levels.

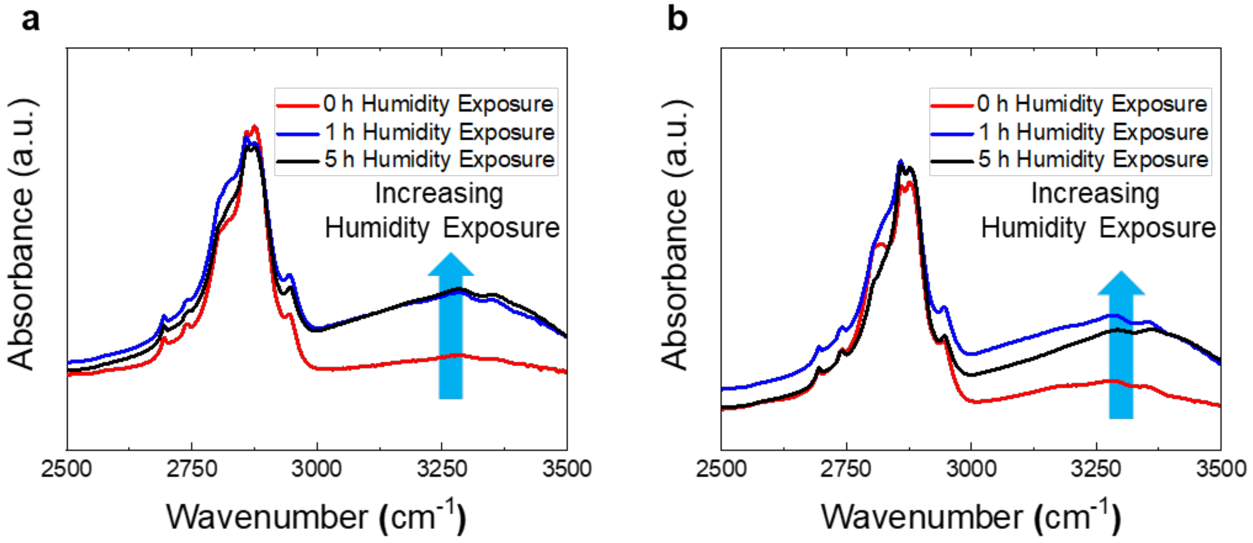

Figure S2. (a) FTIR spectra of a 1:1 PEO:PEI polymer blend film after being annealed and dried under vacuum to remove any excess solvent and exposed to 40% relative humidity at room temperature for up to 5 hours. **(b)** FTIR spectra of a 3:1 PEO:PEI polymer blend film after being annealed and dried under vacuum to remove any excess solvent and exposed to 40% relative humidity at room temperature for up to 5 hours. The broad OH stretch is observed at 3300 cm^{-1}.
